# Immunohistochemical Analysis of the Role of Hemocytin in Nodule Formation in the Larvae of the Silkworm, *Bombyx mori*

**DOI:** 10.1673/031.013.12501

**Published:** 2013-11-05

**Authors:** Ikuyo Arai, Masayuki Ohta, Asahi Suzuki, Shiho Tanaka, Yasutaka Yoshizawa, Ryoichi Sato

**Affiliations:** Graduate School of Bio-Applications and Systems Engineering, Tokyo University of Agriculture and Technology, Koganei 2-24-16, Tokyo 184-8588, Japan

**Keywords:** cellular immunity, granulocyte, innate immunity, von Willebrand factor

## Abstract

Hemocytin, a multidomain protein from *Bombyx mori* L. (Lepidoptera: Bombycidae), is an ortholog of von Willebrand factor and is expected to be a major mediator of hemocyte aggregation. Antiserum was generated against hemocytin, and immune staining of hemocytes, hemolymph, and nodules was performed. Hemocytin was observed in steady-state hemocytes but not in plasma, even after bacterial injection. When hemolymph was smeared on glass slides, hemocytin-containing fibrous structures formed a cellular network mainly consisting of granulocytes and oenocytoids. Hemocytin was stained only in the granules of the granulocytes. When nodule-like aggregates formed 30 sec after bacterial injection, both granulocytes and bacterial cells were observed binding to hemocytin-containing fibrous structures. When nodule sections were stained with antiserum, hemocytin was seen in the matrix of the nodules surrounding the hemocytes. These data suggest that hemocytin plays a major role in nodule formation as a component of the sticky fibrous structure exocytosed from granulocytes.

## Introduction

Innate immunity plays a key role in the defense against microbial infection in invertebrates, which lack an acquired immune system. In insects, pathogens recognized as “non-self” are eliminated by both humoral and cellular responses (Vilmos and Kurucz 1998). The cellular responses, which are mediated by hemocytes, include phagocytosis, encapsulation, and nodule formation (Hoffmann and Reichhart 2002; Cerenius and Soderhall 2004). Nodule formation is a prompt response to clear microorganisms from the hemocoel. It consists of the aggregation and subsequent melanization of hemocytes and microorganisms (Ratcliffe and Rowley 1981). In the humoral responses, humoral factors, including antimicrobial peptides, prophenoloxidase (proPO), which regulates melanization, serine proteases, serine protease inhibitors, serine protease homologs, and lectins, are involved (Vilmos et al. 1998; Kanost et al. 2004; Gupta and Surolia 2007). Pathogen recognition by insect innate immunity involves patternrecognition proteins, including lectins, that bind to pathogen-associated molecular patterns mainly found on the cellular surfaces of microorganisms (Janway et al. 1989; Medzhitov et al. 2002).

Larvae of the silkworm, *Bombyx mori* L. (Lepidoptera: Bombycidae), can scavenge as many as 106 bacterial cells from the hemocoel, and this immune function seems to be largely dependent on nodule formation (Koizumi et al. 1999a). Also, in other insects, nodule formation is considered a major process in the early immune reaction that contributes to the clearance of microorganisms from the hemocoel (Rowley and Ratcliffe 1989; Ratcliffe and Gagen 1997). In contrast, antimicrobial peptides might work at a later stage of infection, since their production and their concentration in the hemolymph increase after bacterial invasion (Imamura et al. 1999). The proPO-activating system for melanization, the most well-known immune response in insects, was found to not always be important for bacterial immunity in *Drosophila melanogaster* (Leclerc et al. 2006; Tang et al. 2006; Schnitger et al. 2007). Furthermore, more than 30 min is required for melanization after the injection of microorganisms. Based on these studies, nodule formation seems to be an important event in the early immune response.

Soon after bacterial injection into the hemocoel, granulocytes release sticky material, and the hemocytes and bacterial cells clump together, resulting in the formation of nodules (Ratcliffe and Gagen 1977; Rowley and Ratcliffe 1981). Few studies have been conducted on the regulation of this aggregation. C-type lectins with two dissimilar carbohydrate- recognition domains, BmLBP and BmMBP, were reported to play an important role in nodule formation (Koizumi et al. 1999b; Watanabe et al. 2006). Additionally, a protein with a reeler domain, Noduler, in *Antheraea mylitta* reportedly regulates nodule formation by binding hemocytes and invading bacteria to each other due to its sticky properties (Gandhe et al. 2007).

The huge multidomain protein hemocytin, which is an ortholog of the human von Willebrand factor and has domains homologous to coagulation factor VIII, was identified in *B. mori* (Kotani et al. 1995). It is involved in platelet aggregation, and coagulation factor VIII participates in blood coagulation in mammals (Toole et al. 1984; Jenny et al. 1987). Furthermore, a study reported that hemolymph taken from genetically modified *D. melanogaster* in which the discoidin domain of hemolectin, an ortholog of hemocytin, was knocked out did not aggregate bacteria (Leasch et al. 2007). From these results, hemocytin is expected to function in the hemolymph coagulation or hemocyte aggregation processes, such as nodule formation, in *B. mori*. In this study, antiserum was generated against hemocytin, and immunostaining of hemocytes, hemolymph, and nodules was conducted to determine the location of hemocytin and its role in nodule formation.

## Materials and Methods

### Laboratory animals

Silkworms, *B. mori* (Kinsyu × Showa), were reared on an artificial diet (Silkmate, Nihonnosanko) containing chloramphenicol at 25° C. Fifth-instar larvae 3–4 days after molting were sterilized by dipping them in 70% ethanol and then used for experiments.

### Preparation of bacterial cells

Gram-positive bacteria, *Micrococcus luteus* IAM1056, (Micrococcales: Micrococcaceae) and Gram-negative bacteria, *Escherichia coli* K-12W3110 (Enterobacteriales: Enterobacteriaceae) were cultured in LB medium (10 g peptone, 5 g yeast extract, 5 g NaCl, and 1 L distilled water). Bacterial cells grown in the logarithmic phase were collected by centrifugation at 8000 rpm for 20 min at 4° C, washed twice with insect physiological saline (150 mM NaCl and 5 mM KCl, pH 6.8), and fixed in 4% formaldehyde with gentle shaking for 1 hour. The fixed cells were collected by centrifugation at 3300 rpm for 20 min at 4° C and washed five times with insect saline.

### Production of anti-hemocytin antiserum

The cDNA corresponding to a partial fragment (N1273–S1569) of Hemocytin was inserted into an expression vector, pGEX-4T-3 (GE Healthcare, http://www.gehealthcare.com) to make a fusion protein of GST and Hemocytin (N_1273_–S_1569_). *E. coli* BL21 cells were transformed with this expression vector. Overnight cultures of transformed BL21 cells were diluted with fresh LB medium containing 100 mg/ml ampicillin, and the cells were grown at 37° C. Production of the fusion proteins was induced by the addition of isopropyl-β-Dthiogalactoside to a final concentration of 1 mM. The bacterial cells were harvested by centrifugation and sonicated to release the inclusion bodies, which were denatured with 10 mM dithiothreitol and 25 mM NaOH and dialyzed in phosphate buffered saline (10 mM sodium phosphate buffer and 145 mM NaCl, pH 7.4). The fusion protein was prepared by preparative electrophoresis, and rabbit antiserum to the fusion protein was raised by Protein Purify, Ltd.

### Bacterial challenge

The density of a prepared suspension containing 4% formaldehyde-fixed bacterial cells was adjusted to OD_600_ 0.8. Bacterial cells were stained with Coomassie Brilliant Blue R250 (CBB) and washed five times with insect saline. The bacterial suspension was centrifuged at 8000 rpm for 10 min at 4° C and washed. Then the precipitate was resuspended in 100 µl of insect saline. For the bacterial challenge, 10 µl of CBB-stained cells was injected into the hemocoel of the *B. mori* larvae using a microliter syringe (Hamilton, http://www.hamiltoncompany.com). The bacteria were also labeled with fluorescein-4- isothiocyanate (Dojindo Molecular Technologies, Inc., http://www.dojindo.com) and washed five times with PBS. For the bacterial challenge, 10 µl of fluorescein-labeled cells was injected into the hemocoel.

### Western blotting of hemocytin

*B. mori* fifth-instar larvae were injected as described above and punctured with a sterile needle. Hemolymph samples from non- injected larvae and bacteria-injected larvae were collected in insect saline mixed with benzamidine (10 mM) and dithiothreitol (DTT) (4 mM) and centrifuged at 800 g for 20 min at 4° C. The supernatant was collected as a plasma fraction. The plasma was mixed with 2 × sample buffer and separated by SDSPAGE (Laemmi 1970) using 12.5% polyacrylamide. For western blot analysis, the proteins were transferred onto PVDF membrane. After blocking with 2% BSA, the membrane was incubated with 30000-fold diluted rabbit anti-hemocytin antiserum for one hour. After washing, the membrane was incubated with 10000-fold diluted peroxidaseconjugated goat anti-rabbit IgG (Wako Pure Chemical, http://www.wako-chem.co.jp) for one hour, and then the ECL western blotting detection system (GE Healthcare) was used to detect peroxidase activity.

### Staining of smeared hemocytes

A drop of normal or bacteria-injected hemolymph from *B. mori* fifth-instar larvae was placed on a glass slide as described above. The cells were smeared in the presence or absence of DTT, air-dried, fixed with 4% paraformaldehyde, washed, permeabilized with 0.1% triton X-100, and stained with 1000-fold diluted rabbit anti-hemocytin antiserum. The cells were washed three times in PBS and incubated with 1000-fold diluted Alexa Fluor 488-labeled goat anti-rabbit IgG or Alexa Flour 594-labeled goat anti-rabbit IgG (Invitrogen, http://www.lifetechnologies.com) for one hour. The nuclei of the hemocytes were counter- stained with 4′-6-diamidino-2- phenylindole (DAPI) (Invitrogen) for two min. Fluorescence was observed under an Olympus BX-50 microscope equipped with BX-FLA epi-fluorescence optics, and the images were obtained with an Olympus Camedia SP-350 camera. In another experiment, smeared hemocytes were fixed with MeOH and stained with Giemsa stain solution (Merck, http://www.merck.com) for 30 min. Oenocytoids were identified by their large cell bodies and large nuclei. Granulocytes were identified by their small nuclei and many small granules. Spherulocytes were identified by their non-Giemsa-stained spherules. Plasmatocytes were differentiated from granulocyte by their intensely Giemsa-stained cytoplasm, larger nuclei, and far fewer small granules.

### Preparation of paraffin sections and immunofluorescence Staining

CBB-stained *E. coli* and *M. luteus cells* were injected in to the hemocoel of *B. mori* fifthinstar larvae, and nodules were collected after incubation. Nodules were fixed in 4% paraformaldehyde in PBS for two hours, embedded in paraffin, and sectioned at 8 µm using a Leica RM2125 microtome. Paraffinembedded sections were transferred onto microscope slides, and the paraffin was removed. After blocking with 2% BSA in TBST (20 mM Tris, 0.15 M NaCl, and 0.05% Tween 20 (pH 7.5)), the sections were stained with 1000-fold diluted rabbit anti-hemocytin antiserum for 60 min. Normal rabbit serum was used as a control. Sections were washed three times in TBST and incubated in 1000- fold diluted Alexa Fluor 488-labeled goat anti- rabbit IgG (Invitrogen) for 90 min. The nuclei of hemocytes were counterstained with DAPI for two min.

## Results

### The location of hemocytin in larval hemolymph

A partial fragment (N^1273^–S^1569^) of hemocytin was produced by *E. coli* as a fusion protein connected to glutathione S-transferase and purified by preparative electrophoresis; this fragment was used to raise antiserum in rabbits. To test the specificity of the antiserum, western blotting was performed against the total protein of the recombinant *E. coli*. A single band was detected with a size of 63 kDa, which is the theoretical mass of the fusion protein (data not shown). Therefore, the antiserum was shown to be highly specific to hemocytin and can be used in western blotting and the immunohistochemical analysis of hemocytin.

**Figure 1.**
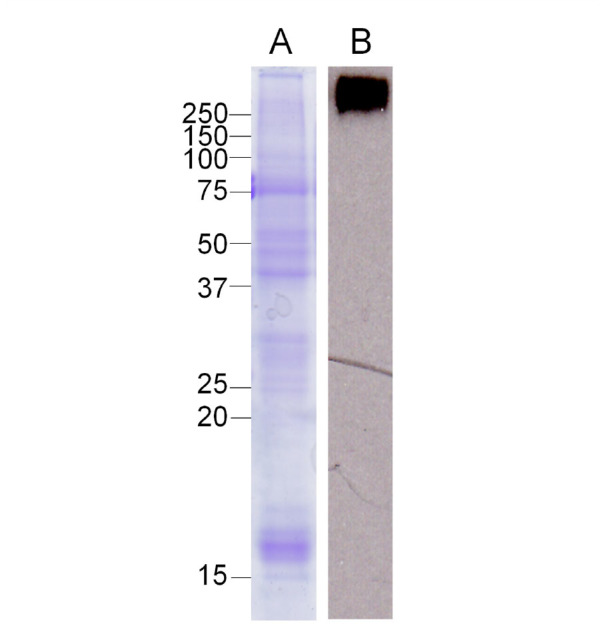
Western blot analysis of hemocytin in hemocytes of *Bombyx mori*. Samples of normal hemocytes were separated by 12.5% SDS-PAGE and stained by Coomassie Brilliant Blue (A). The western blot analysis was conducted (B) as described in the Materials and Methods section. High quality figures are available online.

The expression of hemocytin in hemocytes of *B. mori* larvae increased 10–30 hours after the administration of *E. coli* or *Staphylococcus aureus*, and it was hypothesized to play an important hemocyte-related role in the defense system (Kotani et al. 1995). As a basis for understanding the role of hemocytin in the defense system, we first analyzed the appearance and disappearance of hemocytin in the hemolymph and hemocytes by western blotting after bacterial administration. A single band of the reported size of 280 kDa of the putative mature hemocytin (Kotani et al. 1995) was observed in the steady-state hemocytes (Figure 1B) but not in the steady-state plasma (data not shown). When 10^6^ *E. coli* cells were injected, the plasma hemocytin did not increase even 24 hours after injection (data not shown). These observations suggest that hemocytin accumulates in the hemocytes and does not exist in the plasma as a soluble protein even after bacterial treatment.

### Hemocytin was observed in the fibrous structures exocytosed from the granulocytes and in the granules of the granulocytes

**Figure 2.**
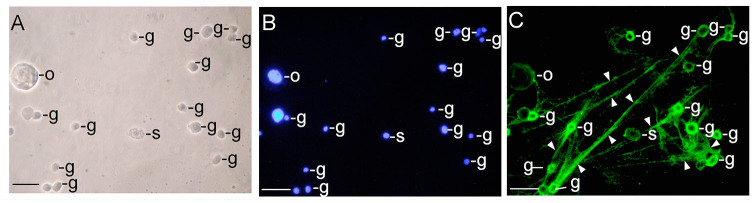
Immunostaining of hemocytin in the smeared hemolymph from *Bombyx mori* larvae. Hemocytes were smeared on glass slides soon after puncture of the larvae, fixed, and stained with anti-hemocytin antiserum in combination with Alexa Fluor 488- labeled secondary antiserum and DAPI as described in the Materials and Methods section. (A) A phase contrast microscopy image of smeared hemocytes is shown. (B) A DAPI-stained image of (A) indicating hemocyte nuclei is shown. (C) An Alexa Fluor 488- stained image of (A) indicating hemocytin is shown. The arrowheads point to the fibrous structures connecting the hemocytes. The scale bars indicate 20 µm. g, granulocyte; o, oenocytoid; s, spherulocyte. High quality figures are available online.

Hemocytin transcripts were detected only in hemocytes by northern blotting (Kotani et al. 1995), and hemocytin did not appear in the plasma even after bacterial challenge. Therefore, we hypothesized that hemocytin is deposited in the granules of hemocytes and exists in the hemolymph in an insoluble form, even though it is secreted by hemocytes in response to bacterial invasion. Hemocytes were observed after staining with antihemocytin antiserum. When the hemocytes were smeared on a glass slide soon after puncturing the untreated larvae with a sterile neeneedle, we observed a network that bound the hemocytes to each other and was composed of a fibrous structure with green fluorescence (Figure 2). Granulocyte cell bodies showed strong fluorescence in a ring shape. The most numerous cells in the network were granulocytes, and inter-granulocyte fibrous structures were broad. In contrast, the cell bodies of oenocytoids, plasmatocytes, and spherulocytes were faintly stained, and the amounts of plasmatocytes and spherulocytes in the network were very low. Only thin, fibrous structures were observed that connected these cells (Figure 2C).

**Figure 3.**
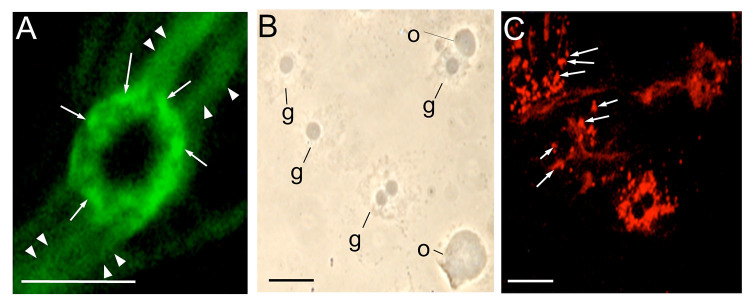
Immunostaining of the accumulated hemocytin in the granules of granulocytes of *Bombyx mori*. After collection in a microfuge tube with a solution of 1 M DTT, hemolymph was smeared on glass slides, fixed, stained with anti-hemocytin antiserum in combination with secondary antiserum labeled with Alexa Fluor 488 (A) or Alexa Flour 594 (C) and observed under fluorescence microscopy (A, C) or phase contrast microscopy (B). The scale bars indicate 10 µm. The arrows point to granules in the granulocyte. The arrowheads indicate fibrous structures. g, granulocyte; o, oenocytoid. High quality figures are available online.

When the granulocytes were viewed under high magnification, stained granules surrounded the nuclei, and the fibrous structures mentioned above were connected to these granules (Figure 3A). Thus, the reason why the cell bodies of granulocytes fluoresced in a ring shape was likely the deposition of hemocytin in the granules. To further confirm the deposition of hemocytin in the granules, the hemocytes were smeared on a glass slide in the presence of 1 M DTT, which inhibits exocytosis of the fibrous structures from granulocytes (Iwahana H, Faculty of Agriculture, Tokyo University of Agriculture and Technology, personal communication). They were then fixed and stained with antihemocytin antiserum. Granules in the granulocytes were stained, but no stained granules were observed in the oenocytoids, plasmatocytes, or spherulocytes (Figure 3C).

**Figure 4.**
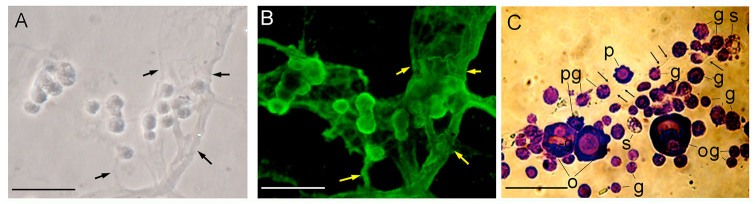
Immunostaining or Giemsa staining of hemocytes aggregated *in vitro*. Hemolymph of *Bombyx mori* larvae was placed on glass slides and left without smearing. The cells were then fixed, stained with anti-hemocytin antiserum in combination with Alexa Fluor 488-labeled secondary antiserum (B) or Giemsa stain (C) as described in the Materials and Methods section and observed under phase contrast microscopy (A, C) or fluorescence microscopy (B). The scale bars indicate 50 µm. The arrows show fibrous structures and broad bands of fibrous structures. g, granulocyte; o, oenocytoid; p, plasmatocyte; s, spherulocyte.High quality figures are available online.

When hemolymph was placed on a glass slide and left without smearing or covering it with a glass coverslip, the granulocytes exocytosed adhesive material with a fibrous structure and eventually aggregated (Figure 4A). This adhesive material, composed of a broad band of fibrous structures, was also stained by antihemocytin antiserum (Figure 4B), suggesting that hemocytin is a component of this adhesive material and that the fibrous structure may be a main factor in forming aggregations, such as the nodules formed when insect larvae are challenged by bacteria. To determine the types of cells comprising it, an aggregate on a glass slide was stained with Giemsa stain. We found that granulocytes were a major compo nent, and oenocytoids were present at the second highest rate (Figure 4C). In contrast, plasmatocytes and spherulocytes were not frequently observed.

### Hemocytin was observed in the hemocyte aggregate formed in vivo upon bacterial challenge

**Figure 5.**
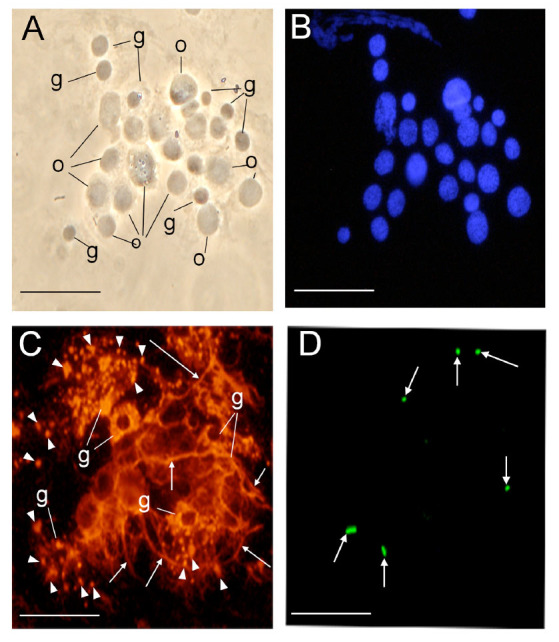
Immunostaining of hemocytin in a small aggregate made *in vivo* after *Escherichia coli* injection. Thirty seconds after fluorescein pre-stained *E. coli* injection, the hemolymph of *Bombyx mori* larvae was placed on glass slides, smeared, fixed, and stained with DAPI (B) or anti-hemocytin antiserum in combination with Alexa Fluor 594-labeled secondary antiserum (C). (A) A phase contrast microscopy image of an aggregate of hemocytes is shown. (B) A DAPI-stained image of (A) indicating hemocyte nuclei is shown. (C) An Alexa Fluor 594-stained image of (A) indicating hemocytin is shown. (D) A fluorescein-stained image of (A) indicating pre-stained *E. coli* cells is shown. The scale bars indicate 20 µm. The arrows show fibrous structures around hemocytes. The arrowheads indicate granules spreading from burst granulocytes. g, granulocyte; o, oenocytoid. High quality figures are available online.

To determine whether hemocytin is involved in nodule formation, *B. mori* fifth-instar larvae were injected with *E. coli* cells previously labeled with fluorescein. Thirty seconds later, hemolymph was smeared on glass slides immediately after puncturing the larvae to prevent the hemocytes from forming aggregates on the glass slides. They were then fixed and stained with anti-hemocytin antiserum in combination with a secondary antiserum labeled with Alexa Fluor 594 or DAPI. Small aggregates composed of granulocytes and oenocytoids were observed (Figure 5A and B). The intercellular joint area of those cells and the fibrous networks around the cells (Figure 5C) were intensely stained with the antiserum. The cytoplasm of the granulocytes and granules, which was smeared from burst granulocytes, was also intensely stained by the fluorescence (Figure 5C). Some oenocytoids had already burst, and only nuclei were observed (Figure 5A). Several fluorescein prestained *E. coli* cells were captured by the aggregate (Figure 5D).

**Figure 6.**
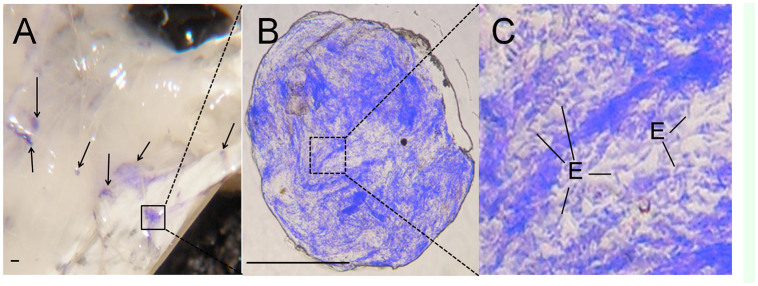
An early stage nodule found 30 seconds after pre-stained *Escherichia coli* injection. *Bombyx mori* fifth-instar larvae were injected with Coomassie Brilliant Blue pre-stained *E. coli* cells and dissected after 30 seconds; the nodules were observed under a stereoscopic microscope (A), dissected, and then observed with a phase contrast microscope (B and C). The scale bars indicate 10 µm. (D) Coomassie Brilliant Blue pre-stained *E. coli* cells in the nodule are shown. E, *E. coli* cell. High quality figures are available online.

The density of the free hemocytes in hemolymph collected 30 sec after *E. coli* injection (106 cells per insect) was determined using a hemocytometer. The density of free hemocytes, with the exception of spherulocytes, was as low as 1% of the level in normal he molymph (data not shown). We speculated that this decrease in hemocytes resulted from the consumption of hemocytes by nodule formation and that the difficulty in finding a nodule at such an early stage was due to the transparency of the nodules. Next, 106 CBBstained *E. coli* cells were injected into larvae, which were dissected 30 seconds after injection to determine whether nodules had already formed. Blue aggregates containing thousands of *E. coli* cells and hemocytes 100–1000 µm in size stuck to the larval tissues (Figure 6). These results indicate that the small aggregate observed in Figure 5 was a small nodule that had not adhered to the tissues and passed through the pore opened by the needle.

### Immunofluorescence staining of hemocytin in a nodule section

**Figure 7.**
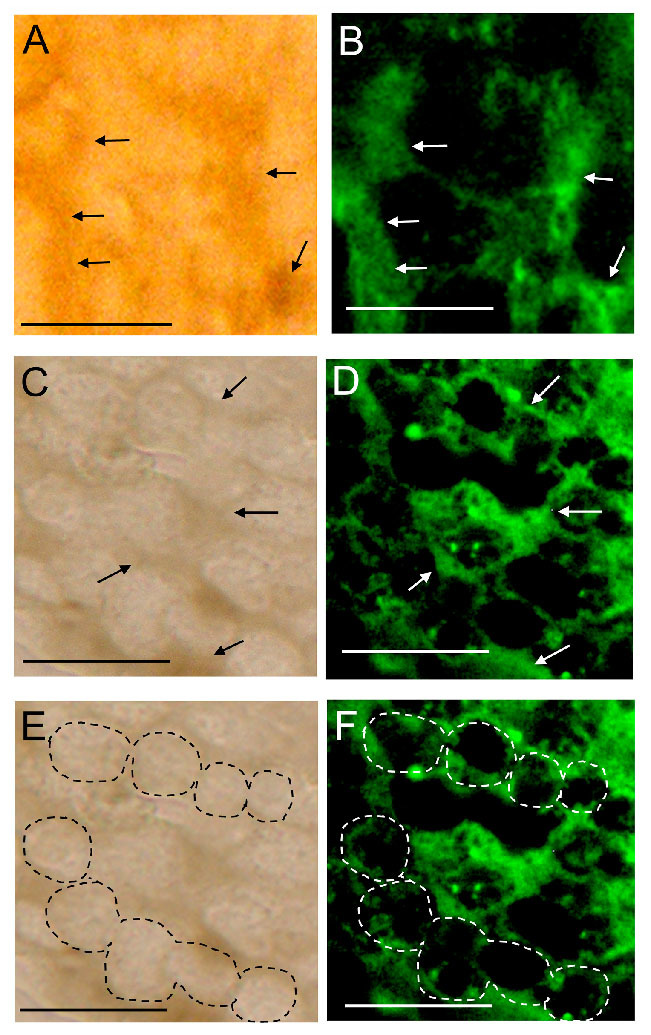
Immunostaining by the anti-*hemocytin* antiserum of the nodule formed in *Bombyx mori* fifth-instar larvae 10 min after injection with *Escherichia coli* cells and 30 min after injection with *Micrococcus luteus*. Paraffin sections of the nodules induced by either *E. coli* (A and B) or *M. luteus* (C–F) injection were stained with anti-hemocytin antiserum in combination with Alexa Fluor 488-labeled secondary antiserum and observed under bright field (A, C, and E) and fluorescence (B, D, and F) microscopy. Under bright field microscopy, the granulocyte cell bodies were observed as bright regions, as indicated by dashed circles in (E), and the matrix was dark, as indicated by the arrows in (A and C). The scale bars indicate 10 µm. High quality figures are available online.

To determine the spatial arrangement of hemocytin in the nodule, nodules formed 10 min after challenge with 10^6^ *E. coli* or *M. luteus* cells were excised and fixed, prepared in paraffin sections, and stained with antihemocytin antiserum. The cell bodies of the hemocytes were confirmed by the existence of nuclei stained with DAPI (data not shown). Hemocyte cell bodies were bright, and the matrix around the cell bodies was relatively dark under bright field microscopy (Figure 7A, C, and E). When the sections were stained with anti-hemocytin antiserum, fluorescence was observed from the matrix and not from the cell bodies (Figure 7B, D, and F). This relationship was similar in nodules formed by injecting *E. coli* or *M. luteus* cells.

## Discussion

### The production of hemocytin by granulocytes and its release through degranulation

Hemolectin, the *D. melanogaster* ortholog of human von Willebrand factor, was reported to accumulate in vesicles of plasmatocytes and crystal cells (Leach et al. 2007), and hemocytin, the *B. mori* ortholog of von Willebrand factor, was reported to be produced in hemocytes (Kotani et al. 1995). In this study, we confirmed that the production of hemocytin was restricted to the granulocytes in *B. mori* and that it accumulated in the granules of granulocytes (Figures 1 and 3). Hemocytincontaining fibrous structures were released from granulocytes in response to stimuli when the hemolymph was smeared on glass slides (Figure 2C). In addition, in nodule-like aggregations, which were observed 30 seconds after the injection of *E. coli*, the cell bodies of the granulocytes were surrounded by a hemocytin- containing matrix (Figure 5C). All of these observations indicate that hemocytin is promptly released from granulocytes in response to stimuli such as bacterial invasion or contact with air. A protein from *Manduca sexta* called M13, which was later renamed scolexin, was reported to induce plasmatocytes and granulocytes to release fibrous structures within one sec (Minnick et al. 1986). M13 may be a mediator that elicits the release of hemocytin-containing fibrous structures by degranulation. Degranulation is reportedly involved in blood coagulation in horseshoe crabs. The activation of a proteaseactivated receptor (PAR) and phospholipase C, as well as the production of inositol-1,4,5- triphosphate (IP3), mediates the degranulation reaction, which is also inhibited by the phospholipase C inhibitor U-73122 (Ariki et al. 2004; Kurata et al. 2006). Recently, U-73122 was shown to inhibit nodule formation in *B. mori* larvae (Suzuki et al. 2012). These data suggest that PLC-mediated degranulation of the hemocytes is involved in nodule formation in *B. mori*.

### Nodule formation depends on network construction, and hemocytin plays a role in nodule formation

Hemocyte—hemocyte connections mediated by a fibrin-like network were observed in association with blood coagulation in crustaceans (Tait et al. 1910). In association with blood coagulation, a fibrin-like network was also described in some other arthropods (Gregoire and Florkin 1950). Likewise, a fibrous network was detected between hemocytes in *B. mori* when the hemolymph was collected on glass slides, and hemocytin was shown to be contained in the fibrous structure (Figure 2C). Hemocytin seems to have a major role in forming the fibrous network, at least *in vitro*. Hemocytin-containing fibrous structures might become entangled with each other and become thicker, resulting in the formation of aggregates of hemocytes, as observed in Figure 4. This mechanism may also function in nodule formation *in vivo*. Since the nodule matrix was heavily stained by anti-hemocytin antiserum, hemocytin seems to be a major component of the matrix (Figure 7).

Granulocytes and oenocytoids were observed with high frequency in the aggregates, and they were entangled in the net of fibrous structures (Figure 4C). Only a few granulocytes and oenocytoids, but many spherulocytes, were detected in the hemolymph collected from *B. mori* larvae previously injected with 10^6^–10^8^ bacterial cells (date not shown). von Willebrand factor reportedly has a D2 domain that has the potential to bind to collagen (Kotani et al. 1995), and hemocytin also has a D2 domain. It is plausible that the granulocytes and oeno cytoids were removed from the hemolymph by being entangled in the net of hemocytincontaining fibrous structures in the nodules. Incidentally, Ratcliffe and Rowley (1976) reported that the nodules observed 24 hours after bacterial injection were melanized and covered with a layer of plasmatocytes. In this study, in contrast to the granulocytes and oenocytoids, few plasmatocytes were observed either in the nodule-like aggregates formed *in vivo* (Figure 5) or the aggregates formed on the glass slides (Figure 4). This result might indicate that the very early stage nodules were composed mainly of granulocytes and oenocytoids based on their binding to the sticky hemocytin-containing fibrous structures; plasmatocytes might cover the nodules later and be regulated by different mechanisms.

In *B*. *mori*, proPO in the nodule was reported to be activated within 10 min of *E. coli* injection around the area where a serine protease homolog, BmSPH1, was present (Sakamoto et al. 2011). This indicates the existence of mechanisms that assemble factors important to the activation of proPO in the nodule. Oenocytoids and plasmatocytes were reported as proPO-producing hemocytes (Ashida et al. 1998), and the binding capacity to oenocytoids of hemocytin-containing fibrous structures might be one of the mechanisms of assembling factors important for melanization in the nodule.

Hemolymph taken from genetically modified *D. melanogaster*, in which the discoidin domain of hemolectin was knocked out, did not have the ability to aggregate bacteria (Leasch et al. 2007). Similarly to hemolectin, hemocytin has a discoidin domain (Leasch et al. 2007). In this study, *E. coli* cells were seen binding to hemocytin-containing fibrous structures (Figure 5D), and the nodules contained thousands of *E. coli* cells (Figure 6). This suggests that hemocytin-containing fibrous structures play an important role in caching bacterial cells in nodules.

Taking all of the above-mentioned observations into consideration, our hypothesis for nodule formation is as follows. Granulocytes promptly respond to bacteria, releasing hemocytin- containing sticky fibrous structures by degranulation. The fibrous structure forms a cellular network consisting mainly of granulocytes and oenocytoids. Bacteria invading into the hemocoel are captured by the network. Due to the flow of hemolymph, the cellular network eventually becomes entangled, and a nodule is generated.

An *Antheraea mylitta* protein with a reeler domain, Noduler, was hypothesized to regulate nodule formation by connecting hemocytes to each other due to its sticky nature. However, the concentration of Noduler in steady-state hemolymph seemed too low to function in such a manner and only started to increase one hour after bacterial challenge (Gandhe et al. 2007). According to our results, the basic form of the nodule is formed within 30 seconds after bacterial injection, and so the upregulation of protein production after bacterial invasion seems to have no role at this primary stage of nodule formation. Additionally, in experiments now being prepared for publication in our laboratory, Noduler in *B. mori* (BmNoduler) was shown to exist in the steady-state hemolymph but at a level too low to function as a binding material to hemocytes and invading bacteria, in contrast to hemocytin. Furthermore, no Noduler was detected by western blotting in nodules excised from *B. mori* hemocoel 15 min after *E. coli* injection. Whether Noduler mediates nodule formation by virtue of its sticky properties with regard to bacteria and hemocytes remains unclear. In contrast, in this study, large amounts of hemocytin were detected in the matrix of nodule (Figure 7A–D). Considering that hemocytin is an ortholog of von Willebrand factor, it may be a major mediator of nodule formation.

## Abbreviations:

CBB,: Coomassie Brilliant Blue R250;
DAPI,: 4′-6-diamidino-2-phenylindole;
DTT,: dithiothreitol;
IPS,: insect physiological saline;
proPO,: prophenoloxidase
